# Alsterpaullone, a Cyclin-Dependent Kinase Inhibitor, Mediated Toxicity in HeLa Cells through Apoptosis-Inducing Effect

**DOI:** 10.1155/2013/602091

**Published:** 2013-03-12

**Authors:** Chunying Cui, Yuji Wang, Yaonan Wang, Ming Zhao, Shiqi Peng

**Affiliations:** ^1^College of Pharmaceutical Sciences, Capital Medical University, Beijing 100069, China; ^2^Medical Experiment and Test Center, Capital Medical University, Beijing 100069, China

## Abstract

Alsterpaullone, a small molecule cyclin-dependent kinase (CDK) inhibitor, regulates the cell cycle progression. Beyond death-inducing properties, we identified the effect of alsterpaullone on cycle procedure and apoptosis of HeLa cell. It was found that alsterpaullone inhibited HeLa cells in a time-dependent (0–72 h) and dose-dependent (0–30 **μ**M) manner. In the presence of alsterpaullone, HeLa cells were arrested in G_2_/M prior to undergoing apoptosis via a mechanism that is involved in the regulation of various antiapoptotic genes, DNA-repair, transcription, and cell cycle progression. Compared to controls, alsterpaullone effectively prevented HeLa cells from entering S-phase. These potential therapeutic efficacies could be correlated with the activation of caspase-3.

## 1. Introduction

Cervical cancer is the third most common cancer among women in the world and has been associated with loss of cell cycle control that normally delays or even arrests proliferation [1, 2]. Cyclin-dependent kinase (Cdk) inhibitors have the potential to induce cell cycle arrest and apoptosis in cancer cells. As one of them, alsterpaullone was found to selectively inhibit Cdk enzymes, especially in Cdk1 [3, 4]. It has been reported that alsterpaullone not only causes cell cycle arrest but also induces the apoptosis of some cancer cells by activation of caspase-9 through perturbation of mitochondrial membrane potential [5–7]. Cdk inhibitors have been shown to possess a cytotoxic effect on tumor cells via cell cycle related proteins and caspase 3 activity. However, this pharmacologic aspect has yet to be studied in relation to alsterpaullone. In this study, we explored the roles of those proteins in the pharmacologic function of alsterpaullone in HeLa cells. In addition, we elucidated the mechanism of cell cycle arrest and apoptosis of HeLa cells treated with alsterpaullone. Our data showed alsterpaullone can inhibit the proliferation of HeLa cells in the dose- and time-dependent manner. Importantly, it induced cell cycle arrest at G_2_/M phase and apoptosis via the regulation of anti-apoptotic proteins (caspase-3) and cell cycle proteins. This finding is significant, since it suggests that alsterpaullone provides a promising chemotherapeutic tool in anticervical cancer arsenal. 

## 2. Materials and Methods

Alsterpaullone was purchased from Sigma-Aldrich (CAS: 237430-03-4). HeLa cell lines were purchased from the Institute of Basic Medical Sciences, Chinese Academy of Medical Sciences. Dulbecco's modified Eagle medium, fetal bovine serum, and trypsin were purchased from HyClone Laboratories Inc., USA. Penicillin and streptomycin were purchased from Sigma Chemical Company, USA. Dimethyl sulfoxide was purchased from AppliChem GmbH Company of Germany. 3-(4,5-Dimethylthiazol-2-yl)-2,5-diphenyl-tetrazolium bromide and acridine orange were purchased from Amresco Company, USA. Protease inhibitor cocktail (1%, Cat No: 539134) was purchased from Merck, USA. All reagents were chemical grade unless otherwise specified.

### 2.1. Cell Culture and Reagents

HeLa cell line was maintained in RPMI-1640 media (GIBCO, Invitrogen Corporation, USA) containing 10% fetal bovine serum (HYCLON, USA), 2 mmol/L L-glutamine (GIBCO, Invitrogen Corporation, USA), 100 U/mL penicillin (GIBCO, Invitrogen Corporation, USA), and 100 *μ*g/mL streptomycin (GIBCO, Invitrogen Corporation, USA). Cells were cultured in an incubator at 37°C under 5% CO_2_ in air. A stock solution of alsterpaullone in DMSO (10 mM) was prepared and diluted to the concentration. The final concentration of DMSO in culture medium was ≤0.3%.

### 2.2. Assessment of Cell Viability (Dose- and Time-Relationship of Alsterpaullone)

HeLa cells (5 × 10^4^/well) were grown in 24-well plates and treated with alsterpaullone (0–30 *μ*M) or DMSO (0.3%, final concentration) to control wells in medium for 72 h. Attached cells were released by a trypsinization and combined with nonadherent cells. After centrifugation, cells were resuspended in PBS and treated with 0.2% trypan blue. Trypan blue excluding cells were counted using a haemocytometer. Experiments were performed in triplicates independently.

For cell growth inhibition, HeLa cells were seeded in 24-well culture plates at a density of 5 × 10^4^/well. At 0, 2, 4, 60, 12, 24, 48 and 72 h 500 *μ*L of alsterpaullone (final concentration: 10 *μ*M and 20 *μ*M) were added into the wells. Cell number and cell viability were determined using haemocytometer and the trypan blue dye exclusion test. Experiments were performed in triplicates independently.

### 2.3. MTT Cytotoxicity Assay

HeLa cells (5 × 10^3^/well) were seeded into 96-well plates and incubated overnight at 37°C. Alsterpaullone was added to cells (in 6 replicates) and incubated for 72 h at 37°C. Stock solution (2 mg/mL) of 3-[4, 5-dimethylthiazol-2-yl]-2,5-diphenyl-tetrazolium bromide (MTT) was prepared in cell media and sterilized via filtration. Media were removed from cells, and 50 *μ*L of MTT solution was then added into each well and incubated in the dark at 37°C for 4 h. MTT solution was removed, and MTT dye of each well was dissolved in 50 *μ*L of DMSO with agitation. The absorbance was measured at 562 nm to determine the IC_50_ (concentration of alsterpaullone which inhibits cell growth by 50%). HeLa cells (5 × 10^3^/well) were seeded in 96-well plates and incubated overnight, pretreated with 50 *μ*L of Z-VAD-FMK (final concentration was 25 *μ*M) for 2 h to block caspase activity, and treated with alsterpaullone for 72 h.

HeLa cells (5 × 10^3^/well) were seeded in 96-well plates and incubated overnight and pretreated with 50 *μ*L Z-VAD-FMK (final concentration was 25 *μ*M) for 2 h to block caspase activity, followed by alsterpaullone treatment for 72 h. Cells were evaluated by MTT assay.

### 2.4. Cell Cycle Analysis and Detection of Apoptosis

Cells were treated with control (0.3% DMSO) and 20 *μ*M alsterpaullone. Both detached and adherent cells were harvested at 0, 12 h, 24 h, 48 h, and 72 h and washed twice with ice-cold phosphate buffered saline (PBS) and then fixed in ice-cold 70% v/v ethanol for more than 2 h. Cells were washed twice in PBS to remove fixative and stained with 1 mL PI (propidium iodide)/Triton X-100 PBS solution with RNase A. After incubation at room temperature for 30 min, cells were filtered through 95 *μ*m pore size nylon mesh. The analyses of cell cycle and apoptosis were analyzed using Flow cytometry. For each sample 10000 events were stored. The fractions of the cells in G_0_/G_1_, S, G_2_/M phases were analyzed using cell cycle analysis software, winMDI v2.8 (Windows Multiple Document Interface for Flow Cytometry) (The Scrips Research Institute, La Jalla, CA, USA).

### 2.5. Protein Extraction and Western Immunoblotting

HeLa cells were cultured with 20 *μ*M alsterpaullone for 0, 2, 4, 6, 12, and 24 h. After incubation, the nonadherent and adherent cells were harvested. The cells were lysed in RIPA lysis buffer (150 mmol/L NaCl, 1% NP-40, 0.5% Deoxycholate, 0.1% SDS, 50 mM pH 7.5 Tris-HCl, 1% protease inhibitor cocktail (Cat no: 539134, Merck)). The protein concentrations in the different samples were determined using the BCA protein assay kit (Pierce, Rockford, USA). Lysates (50 *μ*g) were fractionated by SDS-PAGE using 8%–15% polyacrylamide gels, based upon the expected molecular weight. The resolved proteins were blotted to a nitrocellulose membrane by semi-dry electric transfer, and the membranes were blocked for 1 h in TBST buffer containing 5% blotting-grade non-fat milk. Membranes were incubated with primary antibodies diluted in 0.1% TBST overnight. Primary antibodies against caspase-3 (Cat no: 552785), PARP [Poly(ADP-ribose) Polymerase] (Cat no: 556494), and Mcl-1 (Cat no: 559027) were purchased from BD Pharmingen (BD Biosciences, San Jose, CA, USA). Survivin (Cat no: sc-17779), Bcl-2 (Cat no: sc-7382), p-Rb (Cat no: sc-12901), and p21 (Cat no: sc-817) were purchased from Santa Cruz (Santa Cruz Biotechnology, Inc. CA, USA). Membranes were washed three times in TBST for 10 min each time and then incubated in TBST containing the appropriate horseradish peroxidase conjugated anti-mouse or anti-rabbit secondary antibodies (Amersham Life Science, USA) for 1 h. The membranes were washed three times for 10 min each in TBST. The bound antibody complex was detected using an ECL chemiluminescence reagent and XAR film (Kodak, Japan) according to the manufacturer's instructions (Amersham Life Science, USA). Equal loading of samples was confirmed by probing for *α*-tubulin. 

### 2.6. Statistics

All experiments were performed at least in triplicate. Data were represented as mean ± SD. Student's *t*-test was used for statistical analysis. A *P* value of <0.05 strongly statistically significant, and *P* < 0.01 was considered very statistically significant.

## 3. Results

### 3.1. Alsterpaullone Inhibited the Growth of HeLa Cells in Dose-and Time-Dependent Manner

**Figure 1:**
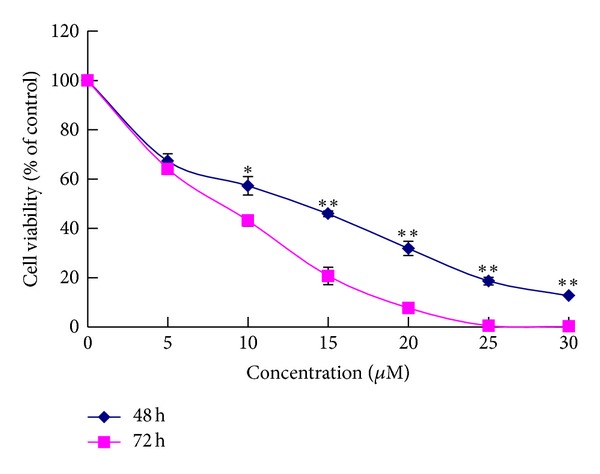
Effects of different concentration of alsterpaullone on the viability in treated HeLa cells. Results are expressed as mean ± SD of three independent experiments. *P < 0.05 for statistical significance; **P < 0.01 for very statistical significance.

**Figure 2:**
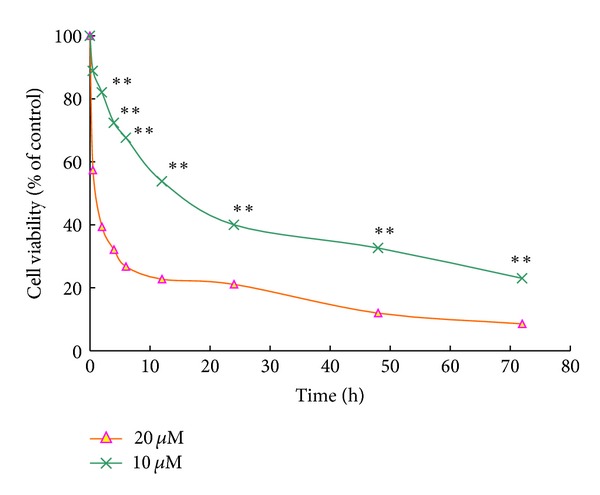
Effects of different time of alsterpaullone on the viability in treated HeLa cells. Results are expressed as mean ± SD of three independent experiments. *P < 0.05 for statistical significance, and **P < 0.01 for very statistical significance.

The growth of HeLa cells was inhibited in a dose-dependent manner after exposures to alsterpaullone for 48 h and 72 h ranging from 0 to 30 *μ*M (Figure 1). The antiproliferation effect was evaluated by measuring the growth rates of HeLa cells seeded at 5 × 10^4^/well in 24-well plates and treated with 10 *μ*M and 20 *μ*M alsterpaullone (Figure 2). Treatment with alsterpaullone caused a time-dependent inhibition of cell growth too. Cytotoxicity was determined by MTT assay following 72 h incubation with alsterpaullone. The IC_50_ value of HeLa cells was 13.80 ± 3.30 *μ*M. Alsterpaullone showed a significant inhibition on HeLa cell proliferation from 10 *μ*M (dose dependent) while from 2 h onwards (time dependent). The results indicate alsterpaullone is a cytotoxic agent in HeLa cells. 

### 3.2. Alsterpaullone-Induced Apoptosis on HeLa is Caspase Dependent

**Figure 3:**
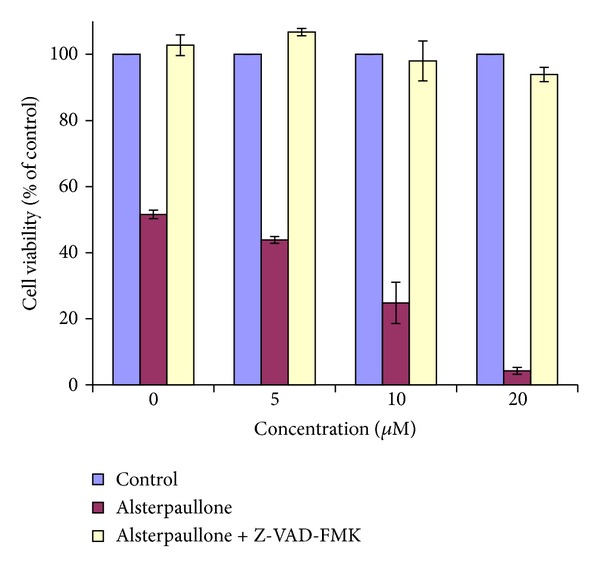
Effects of caspase inhibitor Z-VAD-FMK on apoptotic action induced by 0, 5, 10, and 20 *μ*M alsterpaullone in HeLa cells. Results are given as percentage of viable cells 72 h after the indicated treatment and correspond to mean ± SD.

To explore if the alsterpaullone-induced apoptotic levels are dependent on caspase activation, we cultured cells for 72 h with or without alsterpaullone (0 *μ*M, 5 *μ*M, 10 *μ*M, and 20 *μ*M) in the presence or absence of the general caspase inhibitor VI, Z-VAD-FMK. Viable cells were measured by MTT assay. The apoptotic cells decreased after pretreatment with Z-VAD-FMK, suggesting that Z-VAD-FMK blocks the alsterpaullone-induced apoptosis. The results indicated that alsterpaullone induced apoptosis via caspase-dependent process (Figure 3).

### 3.3. Alsterpaullone Induced G_2_/M Arrest of HeLa Cells

**Figure 4:**
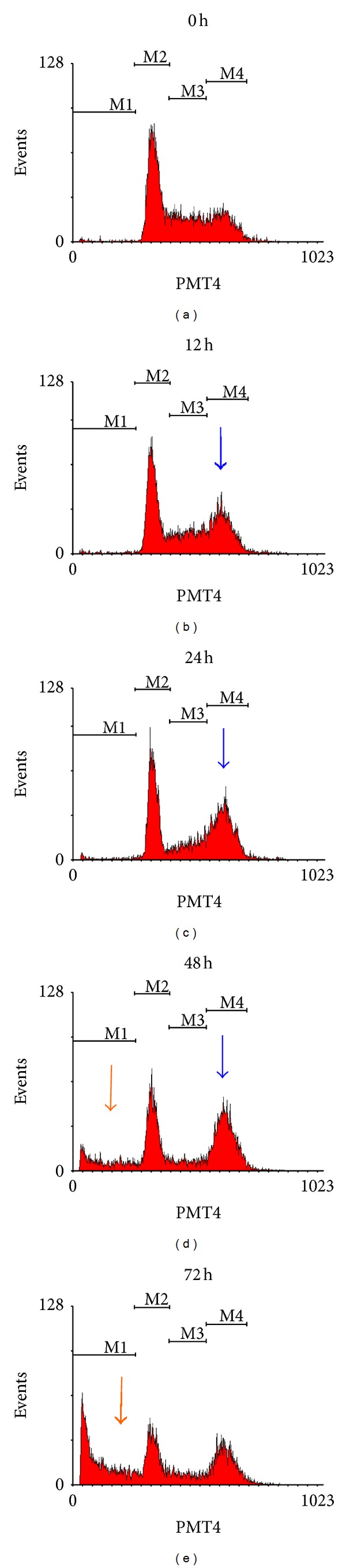
Effects of alsterpaullone on the cell cycle in HeLa cells. Blue arrow: G_2_/M cell cycle arrest; red arrow: sub-G_1_ increase indicating apoptosis occurred. HeLa cells were sensitive to alsterpaullone and arrested in G_2_/M prior to undergoing apoptosis.

 We analyzed the cell cycle profiles of growing HeLa cells exposed to 20 *μ*M alsterpaullone using flow cytometry of propidium iodide stained nuclei. We found the cell cycle arrest occurred at G_2_/M and then apoptosis was induced.  Figure 4 showed a marked increase in the cells with G_2_ contents at 12 h and then the occurrence of significant cell death. The cell cycle G_2_/M arrest persisted and was followed by a sub-G_1_ content increase at 48 h as indicated with cell death. The results indicated the mechanism of antiproliferative effects of alsterpaullone blocked cell cycle progression. 

### 3.4. Apoptotic Proteins in Alsterpaullone-Treated HeLa cells

**Figure 5:**
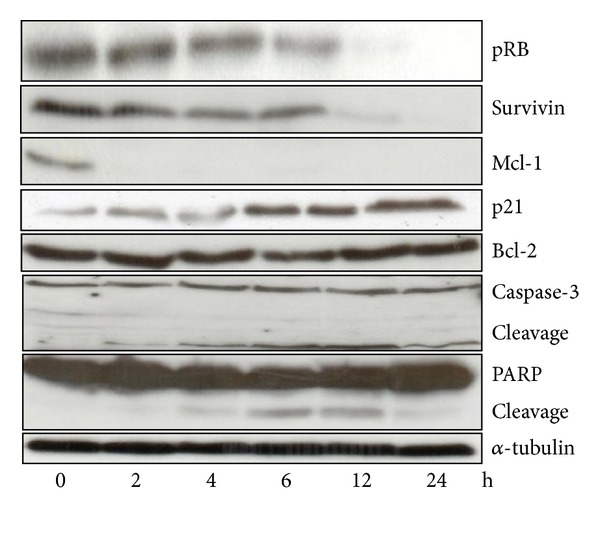
Western blot analysis of apoptotic related proteins in HeLa cells treated with alsterpaullone. HeLa cells were cultured with 20 *μ*M alsterpaullone. Cells were then lysed and protein extracted. The proteins (50 *μ*g) were then subjected to SDS-PAGE immunoblot analysis with the use of antibodies specific for *α*-tubulin, survivin, Mcl-1, PARP, pRB, Bcl-2, and Caspase-3. Cells were also exposed to 0.3% DMSO and showed stable basal levels of the various proteins at the different time points.

To understand the role of alsterpaullone in cervical cancer apoptosis, we performed a time-course study on the apoptotic proteins in 20 *μ*M alsterpaullone treated HeLa cells. As indicated in Figure 5, the cleavage of PARP started at 4 h, while the activation of caspase-3 occurred at 2 h. PARP, a prominent substrate for several caspases, was cleaved in time-dependent fashion indicating the occurrence of apoptosis in alsterpaullone treated cells. Furthermore, direct caspase-3 activation was found in HeLa cells by the cleavage of procaspase-3. Inhibition of caspase activity by the caspase inhibitor Z-VAD-FMK suggests that alsterpaullone induces cell death depending on caspase activity. 

Bcl-2 family proteins play a central role in controlling the mitochondrial pathway, including proteins that suppress apoptosis process (Bcl-2, Mcl-1). In this study a dramatic decline was seen in expression of Mcl-1 which was undetectable at 2 h onwards. The same trend was also observed in survivin but hardly detectable until 24 h. By contrast, the expression of anti-apoptotic protein Bcl-2 was unchanged all the time. These results suggest that the apoptosis induced by alsterpaullone was associated with loss in anti-apoptotic proteins such as Mcl-1 and survivin but not Bcl-2. In alsterpaullone-treated HeLa cells, the levels of pRB kept downregulated overtime whereas p21 upregulated (Figure 5). 

## 4. Discussion

Alsterpaullone, as a Cdk inhibitor, competes with ATP for its binding site on Cdks [7, 8]. Alsterpaullone treatment induced not only cell cycle arrest but also apoptosis in various cell lines [5, 9, 10]. In this study, we showed for the first time that the novel CDK inhibitor, alsterpaullone, inhibited HeLa cell proliferation in a dose- and time-dependent manner. Alsterpaullone induced apoptosis rapidly in HeLa cells by a mechanism that regulates various proteins including anti-apoptotic proteins and cell cycle related proteins.

We found that alsterpaullone exhibited significant cytotoxicity towards HeLa cells, using Flow cytometry and Western blotting: HeLa cells were treated with alsterpaullone arrested in G_2_/M phase prior to apoptosis. This inhibition also led to a drop in S-phase population in HeLa cells and thus disturbed cells' DNA replication [9, 10].

Apoptosis is an important approach through which chemotherapeutic compounds inhibit the growth of tumour cells. Cell death initiated by chemotherapeutic agents usually involves the mitochondrial pathway and releases proapoptotic factors to activate effector caspases, which cause DNA fragmentation and apoptosis [11, 12]. In this study, the results demonstrated the roles of apoptotic proteins in inhibition of alsterpaullone on HeLa cells. Mcl-1 is a short-lived protein because the PEST sequences present with the Bcl-2 family member, and it is an important anti-apoptotic protein [13, 14]. In the current study, we found that Mcl-1 protein was rapidly down-regulated and even undetectable as early as 2 h in HeLa cells. In studies by Lahusen et al., alsterpaullone did not regulate the expression of IAP, a member of XIAP family, in Jurkat cells and MCF10A cells [5, 15]. On the contrary, our results showed alsterpaullone continuously diminished the expression of survivin within 24 h, which is a member of the inhibitors of caspase (IAP) family. Therefore, in order to determine whether p21 protein plays a role in inhibiting cell proliferation, it was measured in alsterpaullone treated HeLa cells using Western blotting. The results showed that p21 protein was up-regulated during 2–24 h. Considering the essential role of p21 in G_0_/G_1_ cell cycle arrest and cleaved caspase-3 for apoptosis induction, we explored the PARP and caspase 3 proteins expression. The results showed the regulation of p21 was significantly earlier than that of caspase 3 and PARP. As such, we speculated p21 was involved in cell cycle arrest, apoptosis, and growth inhibition via activation of caspase-3.

In summary, alsterpaullone can inhibit tumour cell proliferation in a dose-dependent and time-dependent manner and exhibit significant cytotoxicity in HeLa cells. It can induce rapid apoptosis and block cell cycle via regulation of various apoptotic proteins and activation of caspase. The significance behind this in vitro finding is that it suggests the possibility of using alsterpaullone as a new chemotherapeutic agent in the fight against cervical cancer.
